# Comment on Wróblewska-Czajka et al. Outcomes of Boston Keratoprosthesis Type I Implantation in Poland: A Retrospective Study on 118 Patients. *J. Clin. Med.* 2024, *13*, 975

**DOI:** 10.3390/jcm13216496

**Published:** 2024-10-30

**Authors:** Dominique Geoffrion, Mona Harissi-Dagher

**Affiliations:** Department of Ophthalmology, Centre Hospitalier de l’Université de Montréal (CHUM), Université de Montréal, 1051 Sanguinet, D.01.2273, Montreal, QC H2X 3E4, Canada; dominique.geoffrion@umontreal.ca

We read with interest the article “Outcomes of Boston Keratoprosthesis Type I Implantation in Poland: A Retrospective Study on 118 Patients” by Wróblewska-Czajka et al. [1]. The evaluation of the visual outcomes and complications of Boston keratoprosthesis type I (KPro) patients over three postoperative years provided insights into the successes of this method. We commend the authors for comparing the outcomes between the different surgical indications and wish to discuss the findings of this paper.

The study’s findings regarding the improvement in visual acuity post-KPro implantation are significant. They underscore the vital role of such intervention in cases where achieving useful visual acuity through conventional means proves challenging. The statistical significance observed in visual acuity improvement at postoperative intervals provides concrete evidence of the procedure’s efficacy, reaffirming its value in enhancing patients’ quality of life.

Furthermore, the discussion of the diverse outcomes based on different indications for KPro implantation offers critical insights into patient selection criteria. The authors reported that the visual acuity was dependent on the indication for the procedure, with the greatest improvement after one year for patients with ocular burns. Notably, patients with autoimmune diseases showed the worst long-term results and the highest rate of complications, such as corneal melt and retroprosthetic membrane. Our group has shown that KPro eyes with autoimmune diseases had a higher rate of de novo glaucoma development [2], and we would be curious to know if this complication was also more common in this group of patients.

The variation in visual acuity outcomes among patient subgroups not only highlights the complexity of managing corneal diseases, but also emphasizes the importance of tailoring treatment approaches based on individual characteristics and underlying conditions. Adopting a personalized approach is essential to optimize outcomes and ensure the best possible visual rehabilitation for each patient.

The exploration of postoperative complications adds another layer of understanding to the discussion. The identification of glaucoma as a significant complication emphasizes the need for vigilant postoperative management and follow-up care to mitigate its impact on patients’ ocular health. We wish to reiterate that glaucoma surgery ought to be performed as early as possible in KPro eyes with glaucoma and good visual potential [3]. Prompt detection and adequate management of complications may help prevent visual acuity loss beyond three postoperative years.

Similarly, the discussion on corneal melting and retroprosthetic membrane formation sheds light on the multifactorial nature of complications associated with KPro implantation. Patient selection needs to be optimized by considering pre-existing inflammatory disorders for the evaluation of corneal melting risk [4]. Wróblewska-Czajka et al. highlighted the importance of managing such complications as they can in turn be associated with further disorders. Corneal melting in KPro eyes may be managed by repeat KPro, which should be prioritized over other KPro repair methods to maximize the benefits of KPro implantation [5].

Overall, the study represents a good contribution to the field, providing valuable insights into the efficacy, importance of patient selection, and challenges of KPro implantation.
